# Can Beauty be Measured with Photos? A Systematic Review and Meta-Analysis on Static and Dynamic Physical Attractiveness Ratings

**DOI:** 10.5334/irsp.758

**Published:** 2023-04-21

**Authors:** Patrick Kaschel, Lea Hildebrandt

**Affiliations:** 1 University of Wuerzburg, DE

**Keywords:** physical attractiveness, validity, photo ratings, meta-analysis, systematic review

## Abstract

Most studies on physical attractiveness use (static) photos to rate physical attractiveness. This might not reflect how we perceive people in real, dynamic settings. Based on inconsistent previous studies, we conducted a meta-analysis to evaluate the ecological validity of photo-based attractiveness judgements by comparing them to dynamic stimuli ratings. Our literature search resulted in *n* = 46 effect sizes (*k* = 14 studies). Although the overall correlation between ratings of static and dynamic stimuli is high (*r* = 0.70, 95% CI [0.52; 0.81]), heterogeneity between studies is high as well (*Q*(45) = 168.27, *p* < 0.0001 and *I^2^* = 77.71%), which is mostly explained by unreported stimulus quality and within- versus between-rater designs. A Monte Carlo simulation indicated that the small correlations in some previous studies are potentially correlations which had not stabilized yet. Our findings support that the photo-rating method is an ecologically valid approach to assess physical attractiveness.

Attractive people are often viewed favorably and seem to profit from systematic privileges: They receive better school grades (Ritts et al., 1992), benefits at work (Hosoda et al., 2003) and are generally perceived to possess more positive personality characteristics (Eagly et al., 1991). Having such a broad effect, physical attractiveness is a popular research topic—more than 750 empirical articles were published between 2017 and 2019 only^1^—across a variety of disciplines (i.e., anthropology, orthodontics, sexology, psychology, sociology).

As several researchers noted (Kościński, 2009; Morrison et al., 2018; Roberts et al., 2009; Sinko et al., 2018), physical attractiveness is usually assessed by having a variable number of judges rate static stimuli, such as photographs. Due to the popularity of the photo-rating method, one would assume it was well-validated, but only a few studies have compared photo-based ratings to ratings of dynamic stimuli. Although photo-based attractiveness ratings (static) seem face valid for some interpersonal encounters in the real world such as swiping of photos on dating apps, most encounters for which attractiveness effects are being studied are dynamic settings (e.g., at work, grading at school, interpersonal perception in general; see above). Using static, photo-based ratings might not be a valid method to assess how we perceive others’ physical attractiveness in such dynamic, real-life encounters. For example, fluctuating facial expressions (Penton-Voak & Chang, 2008) and body movements, including gait and dance, are only visible in dynamic stimuli and impact attractiveness judgements (Fink et al., 2015). Moreover, perception of facial symmetry, a factor highly related to physical attractiveness, seems to change with movement (Hughes & Aung, 2018). If the photo-based ratings are indeed valid, these dynamic features should not play a big role in the perception of physical attractiveness. Ratings of the same target (i.e., the person depicted) presented as both static (photos) and dynamic (i.e., videos) stimuli should consequently be highly correlated. However, the reported *static-dynamic associations* range from small and non-significant (Lander, 2008; Rubenstein, 2005) over medium and non-significant ones (Penton-Voak & Chang, 2008) to very strong and significant correlations (Brown et al., 1986; Kościński, 2013; Penton-Voak & Chang, 2008). Even within studies, correlations often vary considerably (e.g., from r = 0.11, p > 0.05 to r = 0.81, p < 0.01; Penton-Voak & Chang, 2008). These inconsistent results indicate that it is unclear whether the large number of physical attractiveness findings using the photo method are ecologically valid. What is more, these studies, which directly investigated the relationship between ratings of static and dynamic stimuli, all relied on videos as dynamic stimuli. It is thus not clear whether these (mixed) findings would generalize to live encounters in the real world. Therefore, our main goal was to conduct a systematic review of studies comparing photo-based ratings to video- as well as live-encounter-based ratings.

Besides estimating the size of the correlation between static and dynamic stimuli, we were also interested in explaining the inconsistent static-dynamic associations reported so far. Different potential moderators have been proposed: For example, Roberts et al. (2009) tested the impact of different design features including sex of raters and sex of targets, contextual scenario of the dynamic stimulus, presentation order of both stimulus types and experimental design (within- vs. between-rater designs). They found support for independent effects on the static-dynamic correlation of each of these features. The effect of sex of both raters and targets in their study is consistent with Lander (2008), but Kościński (2013) found no sex differences in the strength of the static-dynamic association. The correlation also seems to depend on the context: It was very high in a mate-choice condition (Roberts et al., 2009) – although Saxton et al. (2009) found a smaller correlation in a highly similar context. Further moderators, according to Roberts et al. (2009), are the order of presentation and experimental design, but these moderators have yet to be tested in other studies. Another potential source of heterogeneity is varying stimulus quality, such as due to the *frozen face effect*: odd static stimuli are potentially created by extracting single frames from videos, which usually capture the depicted persons in a less flattering or blurred pose in the middle of a movement (Post et al., 2012). However, Kościński (2013) found no evidence for a frozen face effect: Correlations of both photo- and frame-based ratings with ratings of dynamic stimuli were highly similar. Taken together, a variety of factors that potentially moderate the static-dynamic association has been proposed, but there is no consistent evidence so far.

In the light of these unclear results, our goal is to evaluate whether the photo rating method, on which most attractiveness research relies, is ecologically valid. This question is increasingly important not only for research but also for everyday life: Nowadays attractiveness ratings based on pictures are especially common (i.e., on dating apps). This also raises the question whether these photo-based first impressions would generalize to a live encounter (i.e., the first date). We also aim to explain why previous findings on this static-dynamic association differ. This has important implications for the field of attractiveness research: If ecological validity were low, research would be based on an artificial construct that does not generalize to physical attractiveness in dynamic, everyday-life social encounters. Whereas previous research on the ecological validity of photo-based ratings only compared photo- and video-based ratings (*static-video correlation*), our review also includes studies comparing photo- and live-impression-based ratings (*static-live correlation*).

We conducted two studies to answer the research questions on ecological validity of photo-based attractiveness ratings and the reasons for contradicting results. The first study is a systematic review and meta-analysis of studies on the static-dynamic association in which both static-video and static-live studies are reviewed. We explicitly included the method of systematic review into our meta-analysis because it allows us to account for the differences in study quality. Specifically, we appraised the strength of evidence per study with an *appraisal score*. This enables us to assess (the combined effect of) a large number of differences between studies systematically (Petticrew & Roberts, 2008: 2–11). This appraisal score is especially useful as a moderator in small meta-analyses with few studies included to assess whether overall study and reporting quality influence the results. The second study is a simulation that aims to inform the interpretation of the results of the meta-analysis. We show that, and explain how, the number of raters, the number of stimuli and factors that affect correlations (Goodwin & Leech, 2006) contributed to conflicting results in previous studies.

## Study 1: Meta-Analysis

The present study was preregistered (https://osf.io/297rk). All extracted study characteristics, the preregistration, the analyses scripts and the PRISMA checklist (Moher et al., 2009) are available in an Open Science Framework repository (https://osf.io/3qwg7/).

### Method

Our analysis followed current guidelines regarding meta-analyses of correlations (Card, 2015; Harrer et al., 2019).

#### Literature Search

We used a four-pronged search strategy. As a first prong, we searched the databases listed on *ProQuest (comprising PsycInfo, PsycArticles and many more; also includes dissertation databases), JSTOR* and *Web of Science Core Collection* with the following search strings:

- for identification of static-video studies: ((ALL=(static OR photo*)) AND (((TI=(motion) OR TI=(movement)) OR TI=(dynamic)) OR TI=(video*)) AND ((TI=(attractive*) OR TI=(beauty*))))
- for static-live studies: ((ALL=(static OR photo*)) AND (TI=(live*) OR TI=(real-live)) AND ((TI=(attractive*) OR TI=(beauty*)))).

The term *ALL* implicates that all fields in the databases were searched, whereas the term *TI* denotes that only title fields were searched. The asterisk acted as a wildcard and the above search string was adopted to the respective search syntax of a database. We first screened all hits and then assessed their eligibility by reading the full texts. As a second prong, we used the search engine *Google Scholar* to identify additional articles in which case we also searched for physical attractiveness outside of the title field to identify even more hidden articles. In the third step, we screened citing and cited studies of our included articles for further studies. As the last search prong, we searched psychfiledrawer.org and osf.io for further grey (i.e., unpublished) literature in addition to the other grey literature databases (e.g., dissertation databases) we had already consulted (e.g., as part of the ProQuest search).

#### Inclusion Criteria

Articles were included that a) were written in English, b) contained primary or secondary analyses, and c) reported static-video or static-live correlations of d) physical attractiveness ratings measured by using a scale (instead of, e.g., eye tracking). If no correlation was available, studies were only included if the data provided in the paper allowed us to calculate the correlation by using standard effect size transformations (e.g., Card, 2015).

During our eligibility assessment, we found studies on *dental* attractiveness and studies that compared two-dimensional (i.e., a static front view) and dynamically rotating three-dimensional (static) images (i.e., animated to turn from the left to the right) of artificial 3D models. We thus added two additional exclusion criteria: First, we decided to only include studies where at least the whole face was visible while physical attractiveness was rated—faces are most important for physical attractiveness ratings, even more important than bodies (Currie & Little, 2009; Peters, Rhodes & Simmons, 2007). Studies in which only a person’s teeth or body were depicted have not been included in the analysis. Second, we only included studies where the video or live stimuli were footage of a real person because our goal is to assess whether a static stimulus is an ecologically valid proxy for the physical attractiveness of a person as encountered in real life.

#### Coding of Effect Sizes and Study Characteristics

We coded effect sizes and study characteristics based on an appraisal form, which was defined and preregistered before the study search. The form is available from https://osf.io/3qwg7/ along with the preregistration and other supplementary materials. The aim of the appraisal form was to collect not only the effect sizes reported for the meta-analysis, but also further details of the studies. These details might help explain differences between study outcomes. Hence, these details were used to evaluate the overall strength of evidence or appraisal score for every single study and for moderator-analysis as part of the meta-analysis.

Accordingly, the appraisal form contains three types of information: The study information, the effect sizes, and potential moderators along with our appraisal rating. Study information included for example the study author, publication year or type of study. Secondly, we coded the effect size information provided, such as type and strength of the correlation. As a coding rule, we extracted all effect sizes available for different subgroups, for example, for male versus female stimuli. Few of the extracted correlations were Kendall’s τ *or* Spearman’s *ρ* correlations. We transformed Kendall’s τ to Pearson’s *r* using the formula in Walker (2003) and did the same for Spearman’s *ρ* with the formula provided in Zimmerman et al. (2003). In studies where correlations were not provided in the text, we extracted effect sizes from figures with the help of *web plot digitizer* (Rohatgi, 2011). Recent studies showed that this tool leads to precise estimates (Burda et al., 2017; Drevon et al., 2017; Valstad et al., 2017).

Finally, the appraisal form assessed moderators and the appraisal score per study. In order to make this assessment, we searched the current literature for differences between studies, that is, potential moderators of the static-dynamic effect. In total, we identified 23 potential moderator variables, which we classified—to improve the clarity of our approach—into two broad categories: 17 *reporting variables* and 6 *threshold variables* (see below). The appraisal form with all 23 potential moderators and the literature suggesting their impact on the static-dynamic correlation is available as Table S1. Importantly, the 23 moderators served two purposes in our study. First, we combined them for the appraisal score; then we used a subset of the potential moderators for meta-analytic moderator analyses to estimate the influence of single moderators. To score the moderator variables and the resulting appraisal score, we judged whether information on each of the 17 reporting variables was reported (which would count as 1) or not (0) and each of the six thresholds could be reached (1) or not (0). Therefore, a study could score 17 + 6 = 23 points as the maximum appraisal score. Importantly, a low score does not mean that a study is of modest quality but only that the publication provides less relevant information and is consequently less well suited to provide reliable evidence for our research question.

The following example will clarify our approach: ‘Experimental Design’ was one of the 23 moderator variables that we coded. If the information provided in an article revealed whether the experimental design of the study was a between- or a within-rater design, this added +1 point to the appraisal score. As the second purpose, we used the ‘Experimental Design’ variable (with the levels ‘within,’ ‘between,’ or ‘unknown’) in moderator analysis to assess whether experimental design is a significant moderator of the overall meta-analytic effect size.

##### Reporting Variables

For the 17 reporting variables of the 23 moderator variables, it was important to have information on each of them reported in primary studies because of their potentially moderating impact. These reporting variables represent details of the design and there is evidence that each of them could moderate the static-dynamic correlation: order of exposure to stimuli (Roberts et al., 2009), sex of raters (Roberts et al., 2009), sex of targets in static and dynamic stimuli (Penton-Voak & Chang, 2008), experimental (between- vs. within-rater) design (Hönekopp, 2006), facial expression in static and dynamic stimuli (Garrido et al., 2017), body region in static and dynamic stimuli (Hönekopp et al., 2007), duration of exposure to the static and dynamic stimuli (Garrido et al., 2017), targets potentially depicted in an odd pose on photos (Post et al., 2012), targets’ angle of view in static and dynamic stimuli which also indicates the gaze direction of targets (Phillips et al., 1992), stimulus context in static and dynamic stimuli (Roberts et al., 2009), and time between exposure to the static and dynamic stimuli (Hönekopp, 2006).

##### Threshold Variables

The six threshold variables are aspects of the design or analysis for which a certain minimal threshold or preferable practice has been suggested in the literature. If this threshold was met or the preferable practice was reported, the appraisal score was increased by one. The threshold variables include both the number of raters per static and dynamic stimulus (Hehman et al., 2018), the corridor of stability for the correlation (Schönbrodt & Perugini, 2013), post-hoc power, dichotomization of ratings (Card, 2015: 21) and a category for other differences between static and dynamic stimuli. The sample size, or number of stimuli, is directly related to the corridor of stability and the power of a study. The corridor of stability is an interval that a correlation would not surpass anymore, on average, even if further observations had been collected (cf. Schönbrodt & Perugini, 2013). For our expected effect size of *r* = 0.6 (see below for derivation of expected effect size) and a corridor of stability with width *w* = 0.2 and 80% confidence-level, *n* > 25 observations were required for the correlation to stabilize (Schönbrodt & Perugini, 2013). This width and confidence-level for the corridor is the most liberal one for which the required sample sizes are reported in Schönbrodt & Perugini (2013). We chose this corridor specification because we expected sample sizes to be small overall in meta-analyzed studies. If we had chosen a stricter specification, it seemed likely that (almost) all studies would miss this criterion, resulting in zero variance on the corridor of stability variable. If the study sample size was large enough for the correlation to stabilize within the corridor, this increased the appraisal score by one (see Table S1 for details). Post-hoc power calculations are controversial because they are often conducted in a circular fashion so that observed power is a direct function of the observed *p*-value (Hoenig & Heisey, 2001). Recent work demonstrated that deriving plausible effect sizes independently from the included studies circumvents this problem and is useful for the comparison of studies (e.g., Brauer et al., 2019). Dichotomization of scores or ratings has been heavily criticized (Irwin & McClelland, 2003; MacCallum et al., 2002; McClelland et al., 2015). Therefore, we scored the use of *un*-dichotomized, continuous data as preferable. The preferable practice in the ‘other differences’ category was that no other differences apart from the necessary experimental manipulation are present. For example, the hair of the target should not be concealed in the static stimulus and be visible in the dynamic stimulus.

Calculation of post-hoc power and the corridor of stability both required estimating the expected effect size of the overall static-dynamic correlation in the meta-analysis. In this sense, the expected effect size can be understood as the expected overall correlation between ratings of the same persons (targets) based on static versus dynamic stimuli. To estimate the true effect size independently of the data, we expect that static and dynamic ratings of the *same* target are highly similar because much of the depicted information in both stimulus types is the same (but see Fink et al., 2015; Hughes & Aung, 2018; Penton-Voak & Chang, 2008). We thus estimate that the different ratings share approximately 75% of variance (*R²* = 0.75), which would amount to a *true* correlation of \[
{r_{true}} = \;\sqrt {.75} = .87
\] . However, such a true effect size is limited by the measurement error and intra-rater variability of each of the measures; in other words, the expected correlations between two different measures would never exceed the correlation between repeated measures of the same variable.

Accordingly, we based our *expected* overall correlation of static and dynamic ratings on the following formula adapted from Hunter and Schmidt (2004: 36), in which a true correlation effect size is penalized by the (retest) reliability of each separate measure: \[
{r_{expected\;}} = \;{r_{true}}*\;\sqrt {{r_{retestStatic}}*\;{r_{retestDynamic}}}
\] . For the retest reliabilities as the second part of the formula, we only found reliabilities for static physical attractiveness ratings: We extracted an average retest-reliability of *r* = 0.73 based on two experiments from Hönekopp (2006). Because this is the only retest-reliability in the physical attractiveness literature that we are aware of and as we expect similar values for the repeated ratings of dynamic stimuli, we used this value for both reliabilities in the formula. Thus, our expected correlation is \[
{r_{expected\;}} = \;.87{\mathrm{\;}}*\;\sqrt {.73} \; = {\mathrm{ }}0.87{\mathrm{ }}*{\mathrm{ }}0.73{\mathrm{ }} = {\mathrm{ }}0.6351
\] which we round to *r_expected_* = 0.6. Based on this calculation, we used *r_expected_* = 0.6 as a rough estimate for the calculation of post-hoc power and the corridor of stability.

##### Moderator Analysis and the Appraisal Score

We conducted different moderator analyses: Initially, we used the appraisal score (which could range from 0 to 23) as a moderator that represented the combined strength of evidence of the studies; subsequently, we used individual moderator variables for which sufficient observations and variance were obtained for further moderator analyses. Although we are aware that some authors (e.g., Card, 2015) argue against using a combined quality measure (like the appraisal score), we chose this combined approach because traditional moderator analyses can easily be underpowered (Borenstein & Higgins, 2013) and, thus, can lead to erroneous conclusions (Valentine et al., 2010), especially if many moderators are present. Because we encountered many (23) moderators to code and expected to find a relatively small number of studies to review, we initially combined those moderators in the appraisal score. In summary, the appraisal score represents not the effect of a single moderator but a summary rating of how many important moderators were controlled for and whether preferable statistical practices were applied in a study. For our case, if effect size and appraisal score were correlated, this would suggest that studies with more robust estimates (higher scores on threshold variables in the appraisal) and/or more transparent reporting (higher scores on reporting variables in the appraisal) produced different estimates, hinting to the fact that effects from studies with a higher appraisal score should be trusted more.

##### Changes during the Coding Process

While coding, we had to exclude the category *indication of presence of factors affecting the ES [effect size]* from the form because we realized that this assessment is too unreliable. The decision to exclude this category was made before we were aware of the final appraisal score per study. Instead of coding it, we now present this issue in the discussion section. We also had to change the category *number of raters per stimulus* by dividing it into separate ones for static and dynamic stimuli. The same split was necessary for the category denoting the type of Likert scale used.

#### Analysis

All analyses were conducted in R version 4.0.3 (R Core Team, 2013) using the packages *metafor* (Viechtbauer, 2010) and *dmetar* (Harrer et al., 2019). Pearson correlations were transformed to Fisher *z* for all analyses and transformed back to Pearson correlations for reporting.

##### Meta-Analysis

We fitted a random-effects model with the restricted maximum-likelihood estimator (REML) and accounted for dependent effect sizes with robust variance estimation (RVE). RVE was shown to result in unbiased estimates and is the preferable approach for dealing with dependent effect sizes in small meta-analyses with k < 25 studies (Moeyaert et al., 2017). Different RVE implementations are available for R; we used the *robust*-function with small-study adjustment from the metafor package because our studies provide different numbers of effect sizes and as we investigated some moderators within studies (Pustejovsky & Viechtbauer, 2017). As a rule of thumb, at least ten studies per moderator are required for meaningful estimates (Borenstein et al., 2011). Thus, we included maximally two moderators into a model. After studies were coded, we found some moderators with enough observations but no variance or, in case of categorical moderators, with highly unequal proportions of observations within the moderator. Moderator analysis is not reasonable in these cases as satisfactory power is rarely achieved (Hempel et al., 2013). Consequently, we refrained from conducting moderator analyses in such cases. Lastly, small-study bias and publication bias were measured by means of a contour-enhanced funnel plot (Peters et al., 2008) and Egger’s regression test (Egger et al., 1997).

### Results

**Figure 1:**
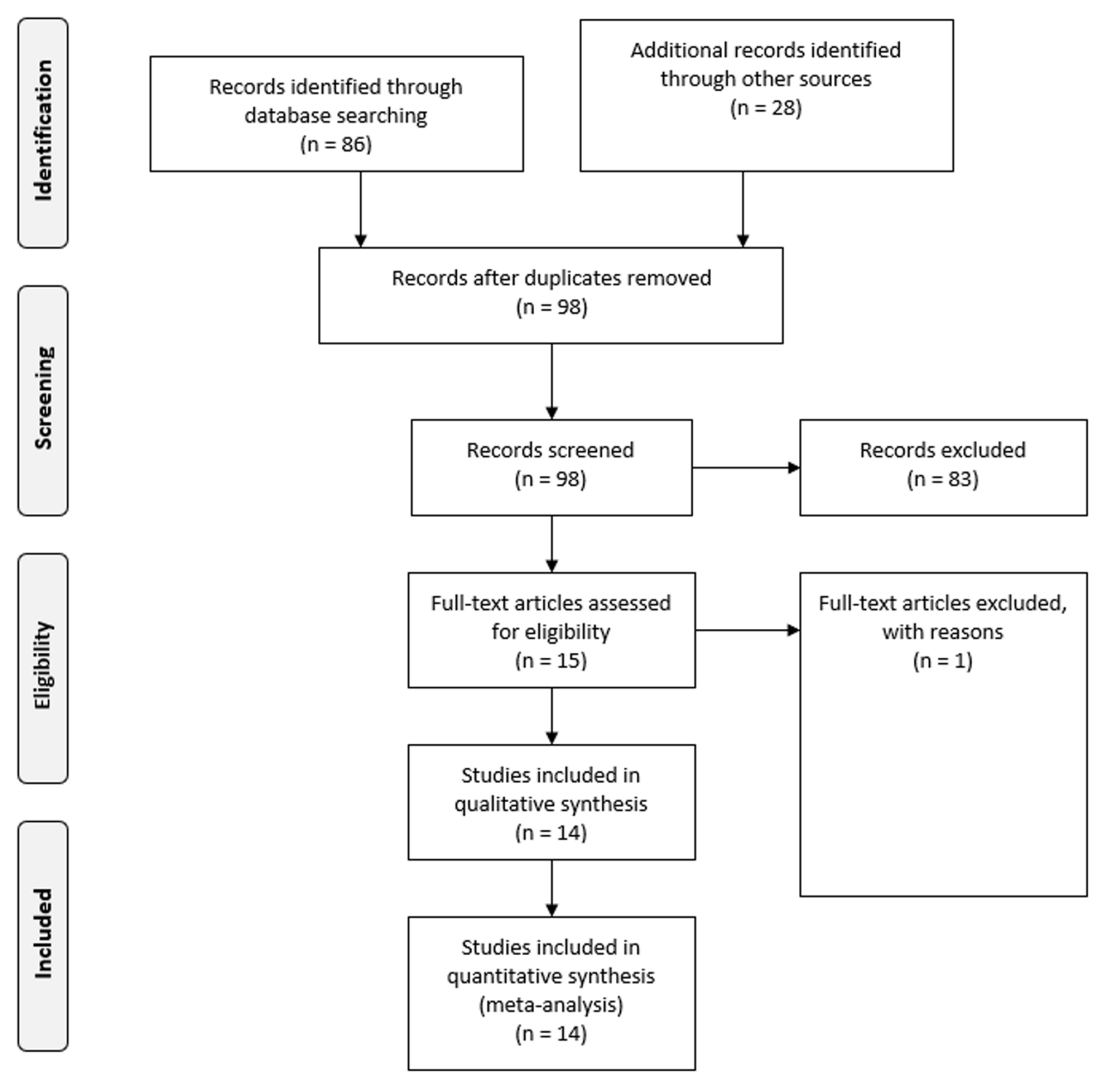
PRISMA flowchart of the literature search process.

All extracted study characteristics and the analyses scripts are available from https://osf.io/3qwg7/. Our literature search identified 15 articles meeting the inclusion criteria. The results of the study search are summarized in a PRISMA flowchart (Figure 1).

One article (Morrison et al., 2018) was excluded during full-text reading because it assessed body attractiveness exclusively. Forty-six effect sizes were extracted from the 14 included articles. Ten of the 14 articles compared static ratings to video-based ratings and four articles compared them to ratings based on a live impression. The group of static-video studies comprised 41 effect sizes and the group of static-live studies five effect sizes. Table 1 presents a summarized version of coded study characteristics whereas the complete coding is available from https://osf.io/3qwg7/ (Table S1).

**Table 1:** Summary of Study Characteristics of Included Effect Sizes.


STUDY	EFFECT SIZE NUMBER PER STUDY	YEAR	DESIGN	STIMULUS MODALITY	SEX OF RATER	PHOTO ODD	DYNAMIC DURATION	DYNAMIC SOUND	PHOTO SEX	DYNAMIC SEX	PHOTO EXPRESSION	DYNAMIC EXPRESSION	PHOTO *N*(RATER)	DYNAMIC *N*(RATER)	PHOTO LIKERT SCALE	DYNAMIC LIKERT SCALE	PHOTO BODY AREA	DYNAMIC BODY AREA	PHOTO CONTEXT	DYNAMIC CONTEXT	N	CORRIDOR OF STABILITY	PEARSON *R*	POWER	*∑* UNREPORTED VARIABLES	*∑* CRITERIA NOT FULLFILLED	APPRAISAL SCORE

Brown et al. (1986)	1	1986	b	V	m/f	no	180	no	m	m	pos	NA	13	4	7	7	hf	h	portrait	interview	115	yes	0.73	1	3	2	18

Brown et al. (1986)	2	1986	b	V	m/f	no	180	no	f	f	pos	NA	13	4	7	7	hf	h	portrait	interview	115	yes	0.71	1	3	2	18

Fischer et al. (1982)	1	1982	b	V	NA	no	450	yes	f	f	NA	NA	5	6	7	7	h	h	NA	interview	21	no	0.78	0.85	6	4	13

Hughes & Aung (2018)	1	2018	b	V	m/f	uk	10	no	m/f	m/f	neut	NA	48	50	10	10	h	h	frame	reciting	46	yes	0.91	1	4	0	19

Koscinski (2013)	1	2013	b	V	m	no	4	no	f	f	neut	mix	10	10	7	7	h	h	portrait	lively	106	yes	0.7	1	0	2	21

Koscinski (2013)	2	2013	b	V	m	no	4	no	f	f	neut	mix	10	10	7	7	h	h	frame	lively	106	yes	0.71	1	0	2	21

Koscinski (2013)	3	2013	b	V	f	no	4	no	m	m	neut	mix	10	10	7	7	h	h	portrait	lively	102	yes	0.6	1	0	2	21

Koscinski (2013)	4	2013	b	V	f	no	4	no	m	m	neut	mix	10	10	7	7	h	h	frame	lively	102	yes	0.69	1	0	2	21

Lander (2008)	1	2008	b	V	m	uk	2	no	m	m	neut	NA	30	30	9	9	h	h	frame	reciting	24	no	0.22	0.90	5	1	17

Lander (2008)	2	2008	b	V	m	uk	2	no	f	f	neut	NA	30	30	9	9	h	h	frame	reciting	24	no	0.36	0.90	5	1	17

Lander (2008)	3	2008	b	V	f	uk	2	no	m	m	neut	NA	30	30	9	9	h	h	frame	reciting	24	no	-0.08	0.90	5	1	17

Lander (2008)	4	2008	b	V	f	uk	2	no	f	f	neut	NA	30	30	9	9	h	h	frame	reciting	24	no	0.54	0.90	5	1	17

Penton-Voak & Chang (2008)	1	2008	b	V	m/f	uk	10	no	f	f	pos	pos	14	14	7	7	h	h	frame	lively	20	no	0.72	0.84	3	3	17

Penton-Voak & Chang (2008)	2	2008	b	V	m/f	uk	10	no	f	f	neut	pos	14	14	7	7	h	h	frame	lively	20	no	0.55	0.84	3	3	17

Penton-Voak & Chang (2008)	3	2008	b	V	m/f	uk	10	no	f	f	pos	neut	14	14	7	7	h	h	frame	reciting	20	no	0.86	0.84	3	3	17

Penton-Voak & Chang (2008)	4	2008	b	V	m/f	uk	10	no	f	f	neut	neut	14	14	7	7	h	h	frame	reciting	20	no	0.80	0.84	3	3	17

Penton-Voak & Chang (2008)	5	2008	b	V	m/f	uk	10	no	m	m	pos	pos	14	14	7	7	h	h	frame	lively	20	no	0.15	0.84	3	3	17

Penton-Voak & Chang (2008)	6	2008	b	V	m/f	uk	10	no	m	m	neut	pos	14	14	7	7	h	h	frame	lively	20	no	0.12	0.84	3	3	17

Penton-Voak & Chang (2008)	7	2008	b	V	m/f	uk	10	no	m	m	pos	neut	14	14	7	7	h	h	frame	reciting	20	no	0.45	0.84	3	3	17

Penton-Voak & Chang (2008)	8	2008	b	V	m/f	uk	10	no	m	m	neut	neut	14	14	7	7	h	h	frame	reciting	20	no	0.42	0.84	3	3	17

Rhodes et al. (2011)	1	2011	b	V	f	no	10	no	m	m	neut	mix	13	13	10	10	h	h	frame	reciting	60	yes	0.83	1	0	2	21

Roberts et al. (2009)	1	2009	b	V	m	no	20	no	m	m	neut	NA	24	24	7	7	hw	hw	portrait	interview	20	no	0.88	0.84	2	3	18

Roberts et al. (2009)	2	2009	b	V	f	no	20	no	f	f	neut	NA	24	24	7	7	hw	hw	portrait	interview	20	no	0.78	0.84	2	3	18

Roberts et al. (2009)	3	2009	b	V	m	no	20	no	f	f	neut	NA	24	24	7	7	hw	hw	portrait	interview	20	no	0.76	0.84	2	3	18

Roberts et al. (2009)	4	2009	b	V	f	no	20	no	m	m	neut	NA	24	24	7	7	hw	hw	portrait	interview	20	no	0.74	0.84	2	3	18

Roberts et al. (2009)	5	2009	w	V	m	no	20	no	m	m	neut	NA	24	24	7	7	hw	hw	portrait	interview	20	no	0.89	0.84	3	3	17

Roberts et al. (2009)	6	2009	w	V	f	no	20	no	f	f	neut	NA	24	24	7	7	hw	hw	portrait	interview	20	no	0.79	0.84	3	3	17

Roberts et al. (2009)	7	2009	w	V	m	no	20	no	f	f	neut	NA	24	24	7	7	hw	hw	portrait	interview	20	no	0.79	0.84	3	3	17

Roberts et al. (2009)	8	2009	w	V	f	no	20	no	m	m	neut	NA	24	24	7	7	hw	hw	portrait	interview	20	no	0.79	0.84	3	3	17

Roberts et al. (2009)	9	2009	b	V	m	no	20	no	m	m	neut	NA	24	24	7	7	hw	hw	portrait	interview	20	no	0.82	0.84	2	3	18

Roberts et al. (2009)	10	2009	b	V	f	no	20	no	f	f	neut	NA	24	24	7	7	hw	hw	portrait	interview	20	no	0.82	0.84	2	3	18

Roberts et al. (2009)	11	2009	b	V	m	no	20	no	f	f	neut	NA	24	24	7	7	hw	hw	portrait	interview	20	no	0.87	0.84	2	3	18

Roberts et al. (2009)	12	2009	b	V	f	no	20	no	m	m	neut	NA	24	24	7	7	hw	hw	portrait	interview	20	no	0.82	0.84	2	3	18

Roberts et al. (2009)	13	2009	w	V	m	no	20	no	m	m	neut	NA	24	24	7	7	hw	hw	portrait	interview	20	no	0.84	0.84	3	3	17

Roberts et al. (2009)	14	2009	w	V	f	no	20	no	f	f	neut	NA	24	24	7	7	hw	hw	portrait	interview	20	no	0.84	0.84	3	3	17

Roberts et al. (2009)	15	2009	w	V	m	no	20	no	f	f	neut	NA	24	24	7	7	hw	hw	portrait	interview	20	no	0.90	0.84	3	3	17

Roberts et al. (2009)	16	2009	w	V	f	no	20	no	m	m	neut	NA	24	24	7	7	hw	hw	portrait	interview	20	no	0.85	0.84	3	3	17

Rubenstein (2005)	1	2005	b	V	m/f	uk	10	NA	f	f	neut	neut	35	35	5	5	NA	NA	frame	reciting	48	yes	0.19	1	4	0	19

Rubenstein (2005)	2	2005	b	V	m/f	uk	10	NA	f	f	neut	neut	40	40	5	5	NA	NA	frame	reciting	48	yes	0.21	1	4	0	19

Saxton et al. (2009)	1	2009	b	V	m/f	uk	20	yes	m	m	neut	NA	13	26	7	7	h	hw	portrait	interview	25	yes	0.52	0.91	4	3	16

Saxton et al. (2009)	2	2009	b	V	m/f	uk	20	yes	f	f	neut	NA	13	26	7	7	h	hw	portrait	interview	26	yes	0.66	0.92	4	3	16

Farina et al. (1986)	1	1986	b	L	NA	no	NA	yes	m/f	m/f	NA	NA	4	1	6	5	NA	hf	portrait	interview	49	yes	0.68	1	8	2	13

Farina et al. (1977)	1	1977	b	L	m/f	no	NA	yes	f	f	NA	NA	14	2	6	5	h	hf	portrait	interview	23	no	0.76	0.89	6	4	13

Farina et al. (1977)	2	1977	b	L	m/f	no	NA	yes	f	f	NA	NA	14	2	6	5	h	hf	portrait	interview	30	yes	0.59	0.96	6	2	15

Gunaydin et al. (2017)	1	2017	w	L	m/f	no	1200	yes	f	f	mix	NA	27	27	7	7	h	NA	NA	interview	55	yes	0.59	1	6	3	14

Howells & Shaw (1985)	1	1985	w	L	m/f	no	NA	no	NA	NA	neut	neut	2	2	10	10	h	h	portrait	interview	54	yes	0.67	1	3	2	18


*Note*: Photo = static photo stimuli; dynamic = dynamic video or live stimuli; b = between; w = within; V = static-video; L = static-live; uk = unknown; pos = positive; neut = neutral; mix = mixed; h = head; hw = head to waist; hf = head to feet; duration provided in seconds; body area indicates which body area of the targets was depicted; m = male; f = female; NA = no information available.

Most studies reported the static-dynamic correlation specifically for the sex of the depicted target (*k* = 11). There were similar numbers of effect sizes across female, male, and mixed-sex rater groups (*n_ESf_* = 13, *n_ESm_* = 12 and *n_ESmixed_* = 19).

Most targets on photos showed a neutral facial expression (*n_ES_* = 35), whereas facial expression was most often unknown for dynamic stimuli (*n_ES_* = 30). Photo stimuli were rated by two to 48 raters (*M* = 19.78) and dynamic stimuli by one to 50 (*M* = 19.43). The number of rated stimuli also differed greatly between studies (range [20; 115], *M* = 37.11). Post-hoc power based on the expected correlation of *r* = 0.6 was always higher than 80%. We also found that only 16 out of 46 correlations reached the corridor of stability. This implies that the other 30 correlations (65% of the correlations) were based on insufficient numbers of observations for the correlation to stabilize (based on the expected correlation of *r* = 0.6, a corridor of stability width of *w* = 0.2 and 80% confidence level).

#### Main Analysis: Association Between Ratings of Static and Dynamic Stimuli

**Figure 2:**
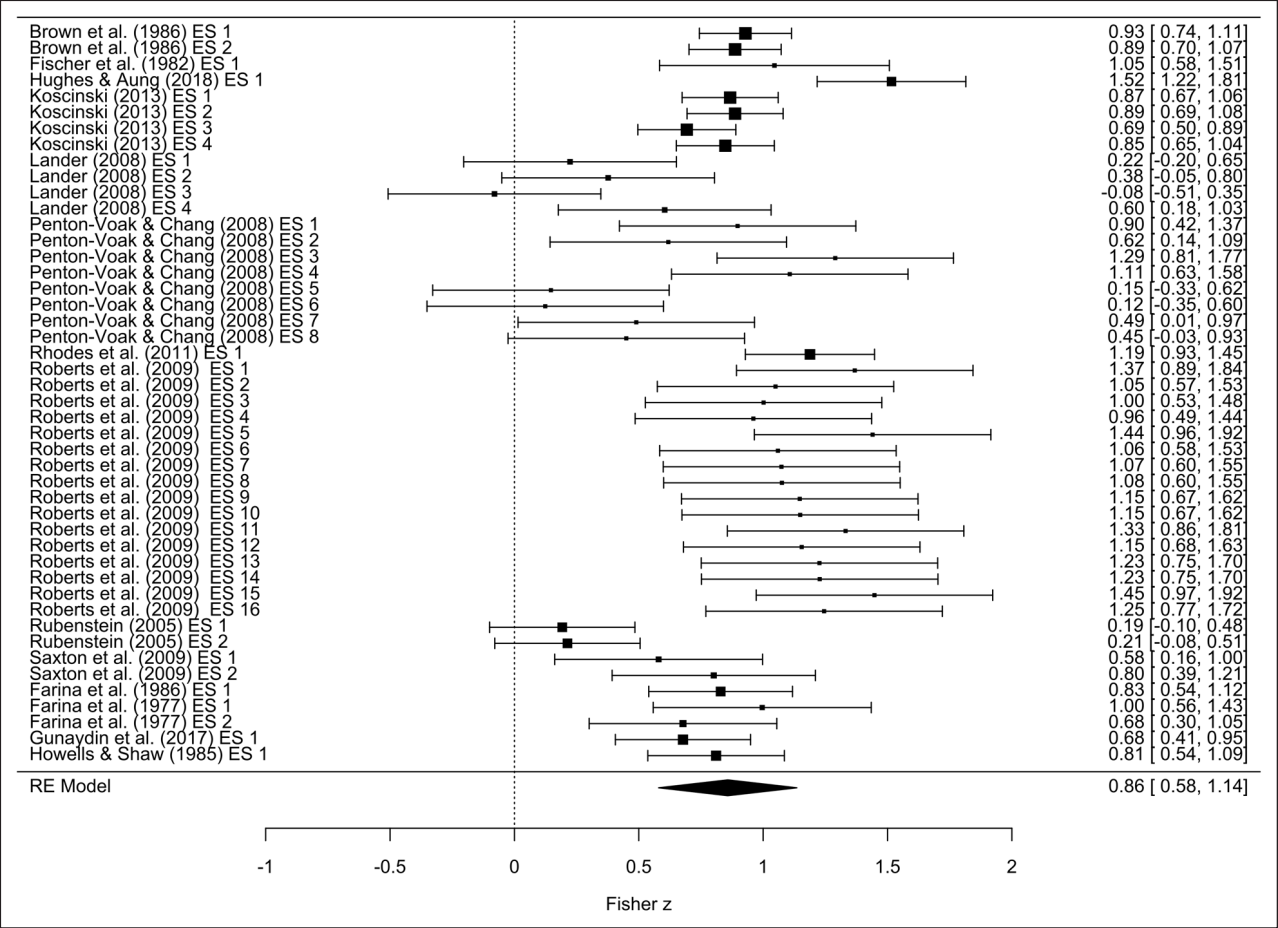
Forest plot of individual effect sizes including their confidence intervals (horizontal lines) and study weights (area of the square); overall effect size estimated with RVE.

The overall correlation between static and dynamic ratings was high (*r* = 0.70, 95% CI [0.52; 0.81], see also Figure 2) with a 95%-prediction interval of PI [0.11; 0.92]. As recommended by Harrer et al. (2019), we applied influential case diagnostics which showed no significant outliers.^2^

**Figure 3:**
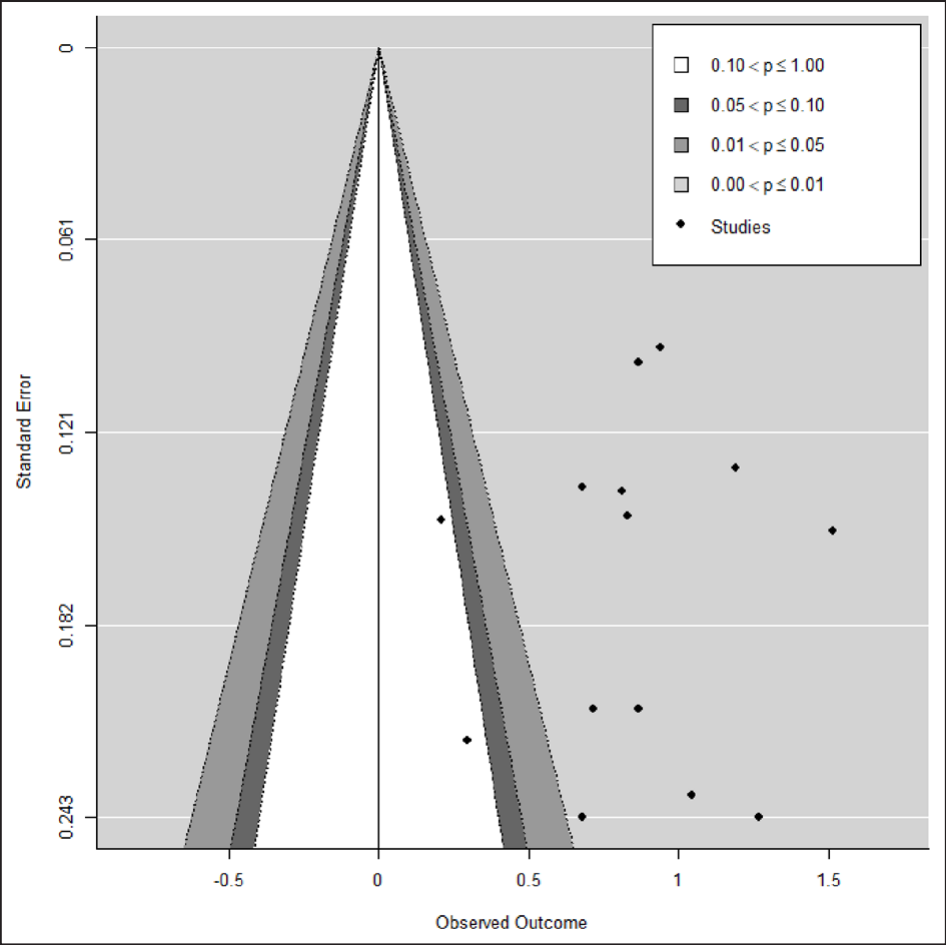
Contour-enhanced funnel plot of aggregated Fisher *z* correlations and their standard errors. The contours display different levels of significance. All of the studies included in this meta-analysis were published.

We also assessed the presence of publication bias in our data. In the contour-enhanced funnel plot of aggregated studies (Figure 3), no accumulation of studies at the significance contours was present. In addition, no asymmetry was apparent, confirmed by Egger’s regression test (*z* = –0.24, *p* = 0.81). Taken together, this implies no evidence of publication bias. High and significant heterogeneity was evident in the effect sizes with 77.71% percent more variation than expected from sampling error (*Q*(45) = 168.27, p < 0.0001; *I^2^* = 77.71%). This indicates that the effect sizes reported in the different studies are rather dissimilar.

#### Moderator Analysis

The appraisal score was used in a first moderator analysis to investigate the combined effect of a variety of potential moderators on the correlations between ratings of static and dynamic stimuli. This analysis shed light on whether studies with lower reporting quality resulted in higher or lower effect sizes. Subsequently, we conducted further moderator analysis with individual moderators to investigate whether these explain the heterogeneous findings.

In particular, we used moderators that a sufficient number of included studies reported, that showed variance between studies and, in case of categorical moderators, did not show highly unequal proportions of observations within the moderator as power would be very low for such cases (Hempel et al., 2013). The included moderators were the body region depicted, the presence of sound, dynamic stimulus modality (live vs. video), face oddity, and experimental design (within- vs. between-rater).

##### Appraisal Score

The overall appraisal score represents a rating of how many important moderators were reported information on in a study (reporting variables) and whether preferable statistical practices are applied (threshold variables). The overall score was moderate to high across studies with *M* = 17.39 out of 23 points and a range from 13 to 21. On average, *M* = 3.15 out of 17 potentially important moderators were unknown (18.54%) and *M* = 2.46 out of six thresholds not fulfilled (40.94%). The appraisal score of the studies was not a significant moderator of the overall effect size (*F*(1, 12) = 0.21, *p* = 0.66).

##### Body Region

Information regarding the depicted body region was available for 42 out of 46 effect sizes. Most of the stimuli depicted either the head only (static: 58.14%, dynamic: 51.16%) or the area from head to waist (static: 37.21%, dynamic: 41.86%). Static and dynamic stimuli depicted the same body region in 85.71% of cases. Whether both stimuli depicted the same body region or not had no significant moderating effect on the overall correlation (*F*(1, 9) = 0.31, *p* = 0.59).

##### Visual and Vocal Attractiveness

For 44 out of 46 effect sizes, the authors indicated whether stimuli were presented with sound (i.e., voice of the target person). Only seven out of 44 stimuli included sound, four of them stemmed from the group of static-live studies. The presence of sound also had no significant moderating effect (*F*(1, 11) = 0.56, *p* = 0.47).

##### Design Issues: Stimulus Modality, Face Oddity and Experimental Design

We assessed whether the effect size is different for static-video (*n* = 41) and static-live studies (*n* = 5). The results showed that modality did not have a significant effect on the overall effect size (*F*(1, 12) = 0.23, *p* = 0.64).

Next, we reinvestigated stimulus quality with a focus on the frozen face effect, which is the finding that single frames extracted from videos are often odd and less flattering, and consequently rated as less attractive than the original dynamic material from which they have been extracted (Post et al., 2012). The judgement whether a stimulus is odd is potentially difficult to make. Therefore, we coded this category conservatively and only as ‘not odd’ if there was clear evidence, that is, the authors confirm in the publication that the frame was not extracted in a moment in which the target showed an odd pose (e.g., half-open eyes or a widely opened mouth; see Post et al., 2012, for examples). For 29 out of 46 effect sizes, it was reported that the underlying static stimuli were not odd. Moderator analysis showed that this was a significant moderator^3^ of the overall effect (*F*(1, 12) = 8.02, *p* = 0.02). The correlation between physical attractiveness ratings of static and dynamic stimuli was considerably higher for studies with reported non-odd photos (*r* = 0.77) than for studies for which this was unknown (*r* = 0.51).

Lastly, we also coded the experimental design of studies and found that 10 effect sizes were from within-rater and 36 from between-rater designs. Moderator analysis showed that design was also a significant moderator (*F*(1, 12) = 7.66, *p* = 0.02). Within-rater designs resulted in higher correlations (*r* = 0.80) than between design studies (*r* = 0.66).

## Study 2: Monte Carlo Simulation

We also conducted a Monte Carlo simulation study using samples of an existing data set with the aim of further explaining the heterogeneity of effect sizes in our meta-analysis. By iteratively calculating the correlation between two sets of attractiveness ratings of the *same* photographs (*static-static* correlation), we assessed the upper bounds for the static-dynamic correlation in the meta-analysis. Specifically, we show which range of correlation is possible for certain numbers of rater and stimuli, even if the same stimuli were assessed in the same modality (static-static). To further explain heterogeneity in the meta-analysis, we demonstrate the importance of correlation-attenuating factors (e.g., the variability in the data) on the size of correlations between static and dynamic stimulus ratings.

### Method

The simulation followed a Monte Carlo simulation approach using an existing dataset of photo-based ratings of attractiveness (Weller et al., 2018). We used this dataset because it includes a large number of photo-based ratings which allows us to draw sufficiently large subsamples. Importantly, our dataset contains only ratings of static photographs and no dynamic ratings, which is why the correlations compare two sets of photo-based ratings from two (independent) groups of raters (between-rater design). Because ratings of stimuli in the same modality (e.g., only photo-based) will most likely be more similar to each other than ratings across different modalities (e.g., photo- vs. video-based), the correlations between photo-based ratings of the same target can be thought of as an upper boundary for the size of static-dynamic correlations. Furthermore, 78% of effect sizes included in the meta-analysis stem from between design studies, which means that the dataset is representative for the majority of studies.

The dataset used includes photo-based physical attractiveness ratings of 48 stimuli from *n* = 1031 subjects on a 7-point Likert scale. Using such a large dataset allowed us to investigate the effect of different numbers of both stimuli and raters on the correlation by repeatedly sampling *S* stimuli rated by *R* raters. We decided to focus on four specific cases that reflected *S* and *R* values of specific studies included in the meta-analysis because they represented edge case rater-stimuli combinations: 1) few raters and few stimuli (Fischer et al., 1982), 2) unequal group sizes of raters and relatively few stimuli (Farina et al., 1977), 3) few raters and a high number of stimuli (Kościński, 2013), and 4) a high number of raters and stimuli (Rubenstein, 2005). For each of the four edge cases, we show the maximum correlation to be expected for this design. The aim of this simulation was to guide the interpretation of the pattern of effect sizes obtained in the meta-analysis. Also, it informs researchers on how many stimuli and raters to use in future research if one has the aim to calculate correlations. For the simulation, we always averaged ratings across raters to obtain an aggregated mean rating per stimulus as it was done in all meta-analyzed studies.

In addition to investigating the largest possible correlation based on this dataset, we also evaluated the impact of factors that affect correlations (Goodwin & Leech, 2006). Goodwin and Leech (2006) present the following six factors: variability in the data, differences in the shapes of distributions, outliers, non-linear association, sample characteristics, and measurement error. We decided to test three of the six factors: variability, shape of the distributions, and outliers. We did not specifically investigate the effects of the other three factors (non-linearity, sample characteristics, and measurement error). Lack of linearity was not assessed in our simulation because it is usually assessed by means of a scatterplot and it is not advisable to inspect the scatterplots of *n_iter_* = 100,000 samples manually. We are also not aware of any suitable statistical test to test for linearity of the relationship. Sample characteristic effects, which predominantly include effects of unintentionally combined subgroups, were already tested for in the moderator analysis. The last factor, measurement error and reliability, is a global factor that affects all studies, and will thus be addressed in detail in the discussion section.

The effects of the remaining three of the six factors affecting correlations (Goodwin & Leech, 2006) were assessed in the following manner: variability was assessed for each of the two variables on which the correlation was based. Instead of testing for differences in the shape of the two distributions on which the correlation is based directly, we assessed whether the individual distributions deviate significantly from normal distributions. We argue that significantly different distributions can naturally occur and pose no problem for the calculation of Pearson’s *r* per se, but non-normal distributions, on the other hand, violate the Pearson correlation assumptions (Field, 2018: 463). For calculation of the number of outliers, we applied the *z* > +/–3.29 criterion (Field, 2018: 340).

### Results

**Figure 4:**
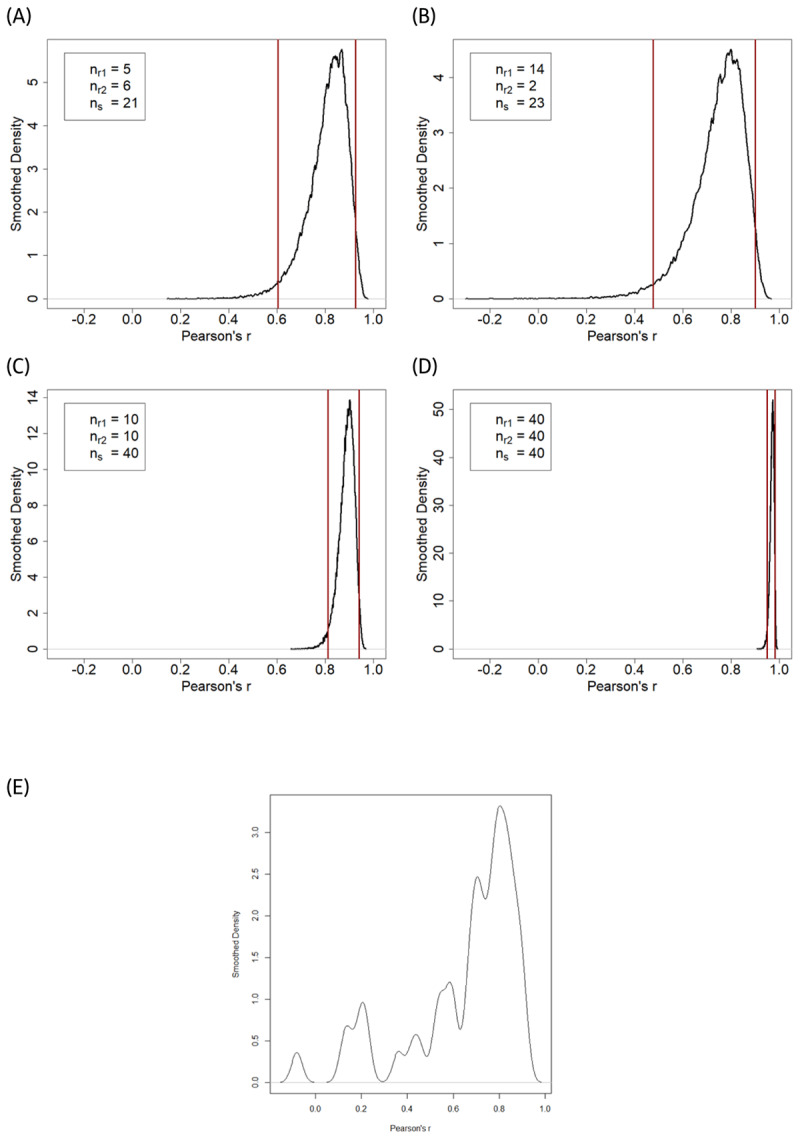
A-D: Probability density of observing different Pearson correlations when different numbers of raters rate different numbers of stimuli. Vertical lines display the 2.5% and 97.5% quantiles. E: plot of the 46 effect sizes from the meta-analyzed studies as a comparison to the simulated edge case distributions; *n_r1_* and *n_r2_* are the number of raters in the two groups; *n_s_* is the number of stimuli.

Our results show that the variability of correlations between two sets of ratings of the same stimuli in the same modality (photo-photo) varies strongly depending on the number of stimuli and the number of raters. For the first two of the four edge case designs with relatively small (Figure 4A) or unequal (Figure 4B) numbers of raters and a small stimulus set, Pearson’s *r* is unstable with a broad range of [0.18; 0.97], or respectively [–0.21; 0.96].

Even though most correlations are high, several remarkably small correlations are present for the first two designs. The correlations vary within a much smaller range of [0.67; 0.96] respectively [0.93; 0.99] for the other two designs with more raters and stimuli (Figure 4, C and D). Notably, a comparison between Figure 4, A-D and Figure 4, E suggests that one should not expect that static-dynamic correlations would be equally high in all meta-analysed studies (Figure 4, E) as some relatively small correlations emerge even for ratings of the same stimuli in the same (static) modality (Figure 4, A-B).

The simulation also showed that extreme outliers based on the criterion of *z* > +/–3.29 are relatively rare for all designs based on the ratings in our dataset (see Table 2). However, a substantial deviation from normal distributions was present in one of the simulated study designs (design B; see Table 2). The variation of the aggregated ratings within a sample of raters and stimuli was also very different between the four edge cases. For the first design, the range of all variances is high [0.14; 2.47] whereas it is relatively small for design four [0.48; 1.09]. This is an important finding, as all these factors have an impact on the size of correlations (Goodwin & Leech, 2006), but the probability of their presence varies between different designs. Our results show that non-normal distributions and more extreme variances (very small or very large) are more likely to be present in designs with fewer raters and stimuli. In accordance with this, very small or very large correlations are much more likely to be present in such designs. These simulations show that, depending on the research design, a variety of correlations are possible—similar to the heterogeneity of effect sizes found in the studies included in the meta-analysis. In future research in which correlations are of interest, researchers may collect larger samples of raters and stimuli to obtain stable correlations. Based on the present data, a number of, for example, *n_r_* >= 10 raters and a number of stimuli *n_s_* >= 40 seems advisable as an absolute minimum (but see Schönbrodt & Perugini, 2013, for a more general investigation of this topic).

**Table 2:** Simulation Results for the Four Edge Case Designs.


DESIGN	MIRRORED STUDY DESIGN	R-R-S-COMBINATION	RANGE OF CORRELATION	CENTERED 95% QUANTILE [0.025; 0.975]	VARIANCE RANGE	NON-NORMAL	OUTLIERS

A	Fischer et al. (1982)	5-6-21	0.18; 0.97	0.6; 0.93	0.14; 2.47	6.76%	0.07%

B	Farina et al. (1977)	14-2-23	–0.21; 0.96	0.47; 0.9	0.03; 4.35	16%	0.3%

C	Koscinski* (2013)	10-10-40	0.67; 0.96	0.82; 0.94	0.28; 1.56	2.62%	0.36%

D	Rubenstein* (2005)	40-40-40	0.93; 0.99	0.95; 0.98	0.48; 1.09	0%	0%


*Note*: R-R-S are the number of raters in group one and group two (R-R), and the number of rated stimuli (S); Monte Carlo samples were collected with *n_iter_* = 100,000 iterations; the outlier column shows the percentage of samples in which any outliers were present and the non-normal column the percentage of non-normal distributions in the samples; * the number of rated stimuli S was reduced to *n_s_* = 40 due to a constrained number of stimuli in our dataset.

## General Discussion

In this meta-analysis and systematic review, we evaluated the ecological validity of photo ratings on which most attractiveness research relies by synthesizing studies that compare those ratings with more naturalistic, dynamic stimuli. We also sought to identify the factors underlying the conflicting results on the static-dynamic correlation.

### Static-Dynamic Association

Even though hundreds or thousands of articles in various disciplines use static photos to study attractiveness, the validity of this method seems poorly studied. This is in line with Kościński (2009) who pointed out that one of the main problems of physical attractiveness research is that a disproportionate number of studies is conducted on a small number of topics while other topics are understudied and resulted in contradictory findings. Although photo-based physical attractiveness ratings are widespread in both research and real-world contexts (i.e., dating apps), it is of particular importance that static ratings generalize to rather dynamic impressions. Seeing a person either on a video or in real-life provides us with a breadth of information (e.g., movement, posture) that might be relevant to a first impression of physical attractiveness. We probably all know from experience the instances when we found someone physically attractive from a brief encounter that we would have found less interesting in a picture. Or, in times of dating apps, we would hope that we find the ‘match’ as physically attractive during the first date as on the pictures provided in the app. Thus, it is crucial that ratings of static stimuli not only generalize to videos, but also to live impressions. While there are few and contradictory static-video studies, even fewer static-live studies exist. Overall, our literature search identified 14 studies comprising 46 effect sizes that allowed us to compare ratings of static and dynamic stimuli. Four of the 14 studies were static-live studies. Only some of the meta-analyzed studies targeted the problem of ecological validity of photo ratings directly, whereas many reported the static-dynamic effect size as ancillary information while focusing on other topics.

Our meta-analysis shows that static and dynamic ratings are strongly related with a Pearson correlation of *r* = 0.70 (95% CI [0.52; 0.81]). This association was stronger than the expected correlation of *r* = 0.6. Despite the fact that dynamic stimuli contain much more information than static stimuli such as fluctuating facial expressions, attractive body movements, or movement-related fluctuations in facial symmetry (Fink et al., 2015; Hughes & Aung, 2018; Penton-Voak & Chang, 2008), ratings from both modalities are highly related. This supports the validity of photo-based attractiveness assessment and justifies using this method in future attractiveness research.

### Interpretation of the Strength of Correlation

What can be concluded from such a high correlation? Some authors argue that even high correlations of *r >* 0.8 still leave much variance unexplained (e.g., Gunaydin et al., 2017; Rhodes et al., 2011). This is both true and to some extent misleading because this argument seems to assume that (almost) 100 percent variance overlap could be achieved (Funder & Ozer, 2019), that is, that knowing one measurement would allow us to perfectly predict the other. However, the correlation (and the variance overlap) is restricted by the error in both measurements (Card, 2015: 131). In fact, even with a perfect true correlation (*r*_true_ = 1), the possible observed correlation can never be higher than the (combined) retest reliability (e.g. \[
{r_{observed\;}} = \;{r_{true}}*\;\sqrt {{r_{retest}}*\;{r_{retest}}} = 1*.73 = \;.73
\] ; using *r_retest_* = 0.73 from Hönekopp, 2006). Along these lines, attenuation correction (Card, 2015: 130–133) of the *observed* meta-analytic effect size of *r_observed_* = 0.70 by the formula \[
{r_{adjusted\;}} = \;{r_{observed}}\;/\;\sqrt {{r_{retest}}*\;{r_{retest}}}
\] shows a corrected correlation of *r_adjusted_* = 0.96. Using another reliability (*r_reliability_* = 0.8417) instead of the retest reliabilities, which we extracted from a study (Kościński, 2013) included in the meta-analysis, results in *r_adjusted_* = 0.83. Both of these adjusted correlations should not necessarily be understood as close approximations of the true correlation but rather indicate that it is unlikely to observe extremely high correlations because all measurements contain error (Bland & Altman, 1996). Overall, it seems that the meta-analytic static-dynamic correlation is high enough to justify measurement of a person’s physical attractiveness by use of static stimuli.

### Heterogeneity-Producing Factors in Previous Studies: Statistical Factors

Another important finding is that in some of the four edge case designs there are correlations that are considerably smaller than the rest. The wide range of correlations found in the simulation study indicates that even with the same underlying true effect, different studies can result in vastly different effect sizes. Rather heterogeneous correlations were indeed found in the studies included in the meta-analysis. As expected, the range of possible correlation is greater with fewer data points collected. Number of raters and stimuli influence the stability of the correlations calculated (see ‘Range of Correlation,’ Table 2). This is also what would be expected based on the simulations by Hehman et al. (2018) and Schönbrodt and Perugini (2013). One explanation for the occurrence of relatively small correlations in each design is that raters with a very different attractiveness taste (Eckes, 2006; Hönekopp, 2006) were present in the two groups of raters. Especially in smaller studies, the impact of diverging raters is substantial. Consequently, if some relatively small correlations emerge even for ratings of the same stimuli in the same (static) modality (Figure 4, A–D), one should not expect that static-dynamic correlations would be equally high in all meta-analysed studies (Figure 4, E).

Ten of the 46 extracted effect sizes were based on a different correlation type than Pearson’s *r*: one study (Penton-Voak & Chang, 2008) with eight effect sizes used Spearman’s *ρ* and another study with two effect sizes (Saxton et al., 2009) reported Kendall’s τ. Importantly, whereas Spearman’s *ρ* is comparable in size to Pearson’s *r*, Kendall’s τ represents a different statistical concept and the values often differ by the factor 1.5 from Pearson’s r’s (Gilpin, 1993; Kendall, 1962: 12). Whereas the absolute size of Spearman’s *ρ* changed little due to transformation to Pearson’s *r* with a maximum deviation of 0.06, transforming Kendall’s τ = 0.35 and τ = 0.46 resulted in Pearson’s correlations of *r* = 0.52 and *r* = 0.66. Thus, one should be careful when comparing correlations across studies without noting their type and possibly transforming them because this can give the impression that a (static-dynamic) correlation is smaller than it truly is.

The fact that non-parametric correlations were used in some cases also suggests that assumptions for the parametric variant were violated. Indeed, one of the two studies using non-parametric correlations mentions assumption-testing and found that their rating distributions were non-normal as soon as data was split for separate analysis by gender of target (Saxton et al., 2009). In addition, Hoekstra et al. (2012) showed that researchers rarely check statistical assumptions before their analysis. Hence, it is likely that more of the 46 effect sizes are affected by unmet statistical assumptions. This is supported by our simulations based on real-world photo ratings which show that, depending on the specific design, about 15% of samples show a non-normal distribution and even extreme outliers of *z* > 3.29 can be present.

### Heterogeneity-Producing Factors in Previous Studies: Moderators of the Static-Dynamic Correlation

Target sex, rater sex, experimental design, facial expression, presence of sound, context of the rating, and the stimulus quality (frozen-face effect) were all proposed as potential moderators of the correlation (Penton-Voak & Chang, 2008; Post et al., 2012; Roberts et al., 2009). We collected information on these and other potential moderators of the static-dynamic correlation (see Table S1 for an overview). Our analysis showed that only differences in the quality of the stimuli (i.e., targets potentially depicted in an odd and unflattering pose in the static condition) and experimental design explain a significant part of heterogeneity of effect sizes between studies. Although we were not able to code directly whether stimuli exhibited the frozen-face effect but rather whether it was explicitly reported that the stimuli were not odd, the frozen-face effect proposed by Post et al. (2012) is one potential explanation for the moderating effect and thus finds further support in our meta-analysis. In addition, static-dynamic effect sizes were considerably higher in within-rater designs with *r* = 0.80 compared to *r* = 0.66 in between-rater designs which is most likely due to raters differing in their perception of attractiveness (Eckes, 2006; Hassebrauck, 1993; Hönekopp, 2006). Therefore, differences in the design features of the studies included, such as varying numbers of raters or stimuli, differences in the quality of the stimuli, as well as using between- versus within-rater designs, greatly contribute to a variety of effect sizes present in the published literature. When accounting for these factors, the overall correlation between static and dynamic stimuli is very high, which indicates that photo-based ratings are an appropriate method to assess physical attractiveness.

### Limitations

Factors that limit our findings include a relatively small number of studies available, especially for the static-live comparison. Moreover, we encountered no study in which both the static and dynamic stimulus showed the target person from head to feet. On the one hand, this makes our results on the static-dynamic association applicable to a sizeable number of studies on physical attractiveness because most research utilizes photographs depicting only head and upper torso (Kościński, 2009). On the other hand, this still leaves the question open whether attractiveness ratings of the whole body, especially if rated from a live impression, result in ratings that are sufficiently associated to static ratings of the head and upper torso. In addition, all of the meta-analyzed studies averaged ratings across raters. Future studies based on unaggregated ratings might provide more insight into idiosyncratic differences in attractiveness perception. Along these lines, it seems promising for our research question to investigate the association of unaggregated ratings of the same targets in different modalities within raters. Lastly, if raw data and rated stimuli had been publicly shared for all meta-analyzed studies, our mission to explain the strong heterogeneity in static-dynamic correlations would have been a much easier one. We hope that this matter will change in the future.

## Conclusion

The high meta-analytic correlation of *r* = 0.70 shows that photo-based judgements are a valid method to measure the attractiveness of a person. Therefore, previous studies, which relied on the photo-rating method, seem ecologically valid. We also demonstrated the impact of stimulus quality (i.e., odd static stimuli, the frozen face effect), the experimental (between- vs. within-rater) design, and other specific design characteristics on the static-dynamic correlation. However, if a static-dynamic correlation is calculated for a study or a subgroup in a study for which the number of raters or number of stimuli is small, a surprisingly extreme (i.e., either small or large) static-dynamic correlation can result. In such a design, the correlation is small or high because the correlation and the ratings on which the correlation is based did not stabilize yet. Designs with higher numbers of raters and stimuli (see Hehman et al., 2018; Schönbrodt & Perugini, 2013, for design recommendations) will result in more reliable correlations. Also, in our review, 65% of the static-dynamic correlations did not achieve the corridor of stability. A Monte Carlo simulation of expected effect size distributions and characteristics for different number of rater and number of stimuli combinations indicates that unmet assumptions for Pearson correlations and other correlation-attenuating factors potentially contributed to small static-dynamic correlations in some studies. As noted in the review and research agenda by Kościński (2009), future research may address the ecological validity of photo ratings more extensively, for example by focusing on the comparability of head-to-feet ratings from live impressions and static photographs of the head and upper torso.

## Data Accessibility Statements

The *R-*Scripts and the data for the meta-analysis are available at https://osf.io/3qwg7/ and the preregistration at https://osf.io/297rk.

## Footnotes

We searched for the exact terms ‘attractiveness’ or ‘beauty’ in the title and ‘physical attractiveness’ as a keyword. Articles with ‘meta-analysis’ or ‘review’ in the title were excluded. For source, we selected *Google Scholar*. The search was conducted with *Harzing’s Publish or Perish* software (Harzing, 1997).

Analyses for influential cases were conducted with the non-robust model because they are not available for RVE. We analyzed the non-robust model and found that the estimated effect size was similar to the RVE estimate (*r* = 0.70, 95% CI [0.55; 0.81]).

Another category in our dataset was applicable for this analysis: the category ‘context’ in which the photo was taken included the two values ‘portrait picture’ and ‘freeze-frame.’ This category and the oddness category were highly correlated (*r* = 0.76, 95% CI [0.60; 0.86]). To prevent problems of collinearity, we only used the oddness category for moderator analysis.
